# Cost-effectiveness analysis of gene-based therapies for patients with spinal muscular atrophy type I in Australia

**DOI:** 10.1007/s00415-022-11319-0

**Published:** 2022-08-18

**Authors:** Tianjiao Wang, Paul Scuffham, Joshua Byrnes, Martin Downes

**Affiliations:** 1 Centre for Applied Health Economics, School of Medicine and Dentistry, Griffith University, Nathan, QLD Australia; 2 Menzies Health Institute Queensland, Griffith University, Gold Coast, QLD Australia

**Keywords:** Cost-effectiveness analysis, Spinal muscular atrophy, Genetic therapy, Rare disease, Nusinersen, Onasemnogene abeparvovec

## Abstract

**Introduction:**

Spinal muscular atrophy (SMA) is an inherited neuromuscular disorder and regarded as one of the most frequent genetic causes of infant mortality. The aim of this study is to develop a cost-effectiveness analysis of AVXS-101 (Onasemnogene Abeparvovec/Zolgensma^®^) and nusinersen (Spinraza^®^) for SMA to inform decision-making on reimbursement policies in Australia.

**Methods:**

A Markov model was developed with five health states to evaluate the costs and effects for patients with SMA Type I from a healthcare system perspective over a time-horizon of 100 years. The model parameters were based on clinical trials, parametric distributions, published literature, and Australian registries. One-way and probabilistic sensitivity analysis were performed to appraise the uncertainties of the parameters in the model. A threshold analysis was conducted to estimate the cost of AVXS-101 of being cost-effective.

**Results:**

The incremental cost-effectiveness ratio (ICER) of AVXS-101 was $1,808,471 per quality-adjusted life year (QALY) and that of nusinersen was $2,772,798 per QALY, compared to standard of care, respectively. The ICER of AVXS-101 was $1,238,288 per QALY compared to nusinersen. The key drivers influencing on ICERs were costs of using treatments and utility values of sitting and walking independently.

**Conclusion:**

Both nusinersen and AVXS-101 resulted in health benefits, but they were not cost-effective with a commonly used willingness-to-pay (WTP) threshold of $50,000 per QALY. Developing high-quality clinical data and exploring appropriate WTP thresholds are critical for decision-making on reimbursement policies in the treatment of rare diseases.

**Supplementary Information:**

The online version contains supplementary material available at 10.1007/s00415-022-11319-0.

## Introduction

Spinal muscular atrophy (SMA) is an inherited neuromuscular disorder, presenting as a progressive muscle weakness and atrophy [1]. It is regarded as one of the most common genetic causes of infant mortality [2], with an estimated incidence of around 1 in 11,000 live births [3, 4].

SMA is an autosomal recessive disorder generally divided into three main subtypes from severe to mild phenotypes: Type I, Type II, and Type III with age of onset of less than 6 months, 6–18 months, and greater than 18 months, respectively [2]. Type 0 (age of onset: prenatal) and Type IV (age of onset: adult) cases can also be observed but they are extremely rare [2]. Type I is expected to account for 60% of all SMA diagnoses [2, 5, 6]. Patients with Type I do not achieve the ability to sit independently and have a median life expectancy of one year with standard of care (SOC) targeting on respiratory symptoms and orthopaedic function decline [2, 7–12], whereas 75–93% of patients with Type II can survive beyond 20 years old [2, 9, 13–15] and patients with Type III can have life expectancy close to the general population [2, 3, 14]. Patients with SMA Type I also experience difficulties in swallowing and/or breathing [16, 17]. Most of the patients rely on permanent assisted ventilation (PAV) at the end of their lives [7].

Although disease management care has improved, patients with Type I SMA treated by SOC do not achieve motor milestones [18]. Nusinersen (Spinraza^®^) as a treatment for SMA, is a survival motor neuron gene 2 (*SMN2*)-directed antisense oligonucleotide and was approved in 2016 by the US Food and Drug Administration [19, 20]. It is currently listed in the Pharmaceutical Benefits Scheme in Australia under a special pricing arrangement with a disclosed price of approximately $110,000 per dose [21]. Patients who take this intervention need four loading doses within two months and maintenance doses every four months thereafter [16].

Onasemnogene abeparvovec-xioi (AVXS-101/Zolgensma^®^), a new genetic replacement therapy for SMA, is a one-time injection for affected children who are less than two years old. AVXS-101 is a non-replicating virus that is used to deliver a functional copy of the survival motor neuron 1 (*SMN1*) gene as an alternative for the defective *SMN1* in the patients’ own cells [16]. The substitution for the defective *SMN1* aims to solve the issues related to the expression of the SMN protein. AVXS-101 was approved by the US Food and Drug Administration in 2019 [22] and was granted market authorisation by the European Commission [23, 24]. In Australia, AVXS-101 was approved by the Therapeutic Goods Association in 2021 but deferred by the Pharmaceutical Benefits Advisory Committee who is responsible for approving reimbursements [25, 26]. The market price of AVXS-101 is US$2.125 million per dose [27] which is unaffordable to the vast majority of affected families. Of note, AVXS-101 is given as a one-off cost whereas nusinersen usually requires regular injections periodically. The high costs of these life-saving drugs necessitate economic evaluations that examine the trade-off between costs and benefits of the drugs’ used in treating SMA.

One systematic review [28] identified six economic evaluations of treating patients with SMA, with three commissioned by health authorities of Canada [29], US [30], and Ireland [31]. Another two were performed by the manufacturers of nusinersen and AVXS-101 [10, 16]. One was conducted by an academic group [32]. Of note, some results of the report by CER Institute [30] were published as a paper in a peer-reviewed journal [33]. Another economic evaluation was recently published for the Dutch population [23]. The ICERs of these studies were heterogeneous, which could be mainly explained by the differences in measuring quality of life and cost, and by the quality of clinical evidence. Additionally, many studies used data from a US context, in which the healthcare system differs from the jurisdictions with publicly funded healthcare systems.

The aim of this study is to examine the cost-effectiveness of AVXS-101 and nusinersen in Australia, for patients with SMA Type I. In addition, a threshold analysis is conducted to estimate the cost of AVXS-101 of being cost-effective with the change of willingness-to-pay (WTP) thresholds.

## Methods

### Model structure

A Markov model was developed with five health states to model the lifespan of patients with SMA Type I, which was built in TreeAge Pro 2022 (TreeAge Software, Williamstown, MA).

**Fig. 1:**
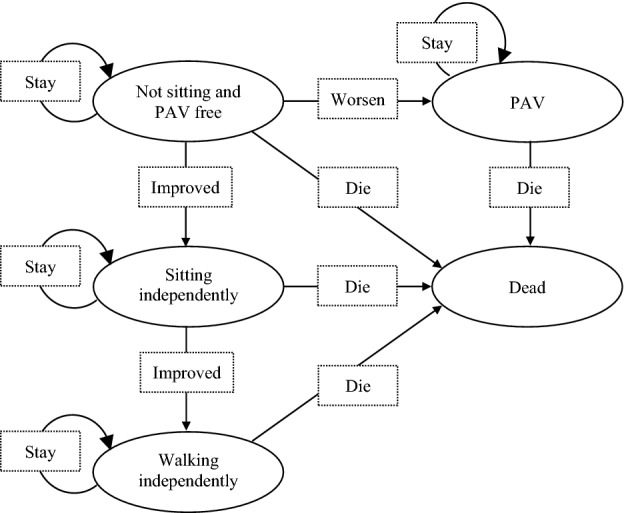
Structure of Markov model in the analysis

Markov models are widely used in economic evaluations of healthcare interventions, which model a cohort of patients over time as they transition between health states (including death) [34, 35]. Information from published economic evaluations for treating patients with SMA Type I was used for building the model [16, 23, 30, 36]. The structure of the model is illustrated in Fig. 1. The health states of “not sitting and PAV free”, “sitting independently”, and “walking independently” reflected the health states corresponding to SMA Type I to III. The health state of “PAV” was defined as patients with SMA Type I in need of permanent assisted ventilation. The health state of “dead” was defined as the absorbing state.

Patients entered the model as infants in the health state of “not sitting and PAV free”. They could transition to “sitting independently” as an improved state, or to “PAV” as a worse state in the next cycle. Patients who achieved the motor milestone of “sitting independently” could transition to “walking independently” in the next cycle. Except for the absorbing health state of “dead”, patients in other health states could either die or stay in their current health states in the next cycle.

The target population was infants born with SMA Type I as the recruited patients in the clinical trials of nusinersen and AVXS-101 [37, 38]. The cost-effectiveness analysis was performed with a healthcare system perspective in the Australian context. Both SOC and nusinersen were used as comparators. A lifetime horizon of 100 years was chosen for this model because patients with SMA Type III could have a life expectancy close to general population [2, 3, 14]. A monthly cycle length was used, which could reflect the details of modelling survival of up to 2-years life expectancy for patients with SMA Type I. All costs and effects were discounted at 5% annually in the base-case analysis as per Australian norm [39, 40]. A half-cycle correction was applied for the model. All the key assumptions are summarised in Table S1 in the supplementary materials.

### Clinical trials

Patients with SMA Type I treated by nusinersen were studied in the ENDEAR (NCT02193074) clinical trial [37]. This is a phase 3, randomised, sham-procedure controlled study recruiting 122 patients with a 13-month follow-up. Since one patient withdrew prior to receiving the treatment, in total, 80 patients in the treatment arm and 41 patients in the control arm received the treatment. At the start of the trial, all the patients were diagnosed with SMA Type I, corresponding to the health state of “not sitting but PAV free” in the model, and the onset of symptoms of those patients were either at six months of age or younger. At the end of the trial, as reported by Finkel et al. [41], 8% of the patients in the treatment arm were improved to “sitting independently”, whereas 0% in the control arm. No patients in both arms reached “walking independently” [41].

Patients with SMA Type I treated by AVXS-101 was studied in a single-arm trial (CL-101) including 12 patients treated with a therapeutic dose of AVXS-101 (2.0 × 10^14^ vg/kg) (NCT02122952) [38], with a two-years follow-up. At the start of the trial, all the patients were diagnosed with SMA Type I, and the onset of symptoms of these patients were up to six months of age. At the end of the trial, 11 of the 12 patients achieved “sitting independently” and two of the 12 patients achieved “walking independently”. No patients requested PAV [42].

### Measurement of utility values

Each health state was assigned a utility value in the model. One published cost-effectiveness study for the Dutch population [23] used the utility values which were similar to another cost-effectiveness study for the American population [16], by mapping utility values from the PedsQL generic score scale to EQ-5D-Y [43]. Compared to these two studies, the CER Institute model [30, 33] for the American population used more conservative utility values taken from Thompson et al. [44] and Tappenden et al. [45], with assumptions of improved utility after treatments. The utility values of the general population in the US were used for “walking independently” in the CER institute model, in which, however, patients would still have some symptoms of SMA. Another published cost-effectiveness study for the Swedish population [10] did not identify the utility values for patients with SMA in the literature, thus the authors used the estimates by five UK clinical experts who reviewed the case study descriptions of SMA [46].

One recently published study of SMA burden quantified the Australian utility values for patients with SMA (from Type I to Type III) [36]. However, the utility values described in this study were not increased as the improvement of motor milestones, which utility value of SMA Type II was lower than that of Type I. The variations of utility values in these studies are shown in Table 1. Given the considerable variations of utility values in the published studies, conservative utility values were used in the base-case analysis of our study. The input parameters can be found in Table 2.

**Table 1:** Comparison of utility values used in the published literature for patients with SMA

Health states	Broekhoff et al. [23] (The Netherlands)	Malone et al. [16] (United States)	Ellis et al. [30] (United States)	Zuluaga-Sanchez et al. [10] (Sweden)	Chambers et al. [36] (Australia)
PAV (permanent assisted ventilation)	0.733	0.730	0.19	− 0.240	NA
Not sitting and PAV free (SMA Type I)	0.733	0.756	0.19 for SOC arm 0.29 for treatment arms	− 0.120	0.104
Sitting independently (SMA Type II)	0.752	0.764	0.6 for SOC arm 0.65 for treatment arms	− 0.040	0.067
Walking independently (SMA Type III)	0.878	0.878	General population (from 0.736 to 0.922)	0.710	0.252

**Table 2:** Input parameters for the base-case analysis (AU dollar, 2020)

Variable	Value	Min	Max	Distribution	Source
Time horizon (years)	100	–	–	Fixed	–
Discount rate: costs	5%	–	–	Fixed	Australian norm
Discount rate: effects	5%	–	–	Fixed	Australian norm
**Drug costs**					
Nusinersen	$110,000	$78,804	$146,349,45	Gamma	PBS
AVXS-101	$3,054,344	$2,138,121	$3,970,796	Gamma	Market price
**Administration and monitoring costs**					
* Nusinersen*					
Intrathecal injection (lumbar puncture into central nervous system)	$77.65	–	–	Gamma	MBS Item 39000
Intrathecal injection (drain cerebrospinal fluid)	$164.4	–	–	Gamma	MBS Item 40018
Specialist	$51.5	–	–	Gamma	MBS Item 2126
Monitor for thrombocytopenia	$16.95	–	–	Gamma	MBS Item 65070
Monitor for renal toxicity	$9.7	–	–	Gamma	MBS Item 66500
Anaesthesia for lumbar puncture	$102	–	–	Gamma	MBS Item 21945
Imaging (ultrasound or fluoroscopy – average cost)	$34.3	–	–	Gamma	MBS Item 55854, 60503
Inpatient cost per diem (routine surgery)	$1,839	–	–	Gamma	NHCDC round 18
Inpatient anaesthesia	$281	–	–	Gamma	NHCDC round 18
*Administration costs of nusinersen*	$2,576.50	–	–	Gamma	–
*AVXS-101*					
Single-dose intravenous infusion	$67.1	–	–	Gamma	MBS Item 13915
Anti-AAV9 diagnostic test	$15.65	–	–	Gamma	MBS Item 68384
Laboratory monitoring	$17.7	–	–	Gamma	MBS Item 66512
Prednisolone	$14.53	–	–	Gamma	PBS 1934T
*Administration costs of AVXS-101*	$114.98	–	–	Gamma	–
*Total treatment costs of nusinersen per dose*^a^	$112,577	$78,804	$146,350	Gamma	–
*Total costs of AVXS-101 per dose*^a^	$3,054,459	$2,138,121	$3,970,796	Gamma	–
**Health state costs**^a^					
Not sitting but PAV free	$23,569	$16,498	$30,640	Gamma	Chambers et al. [36]
Sitting independently	$9,896	$6,927	$12,865	Gamma	Chambers et al. [36]
Walking independently	$6,644	$4,651	$8,637	Gamma	Chambers et al. [36]
PAV	$27,693	$19,385	$36,001	Gamma	Chambers et al. [36] and CER institute [30]
**Utility values (SD)**^b^					
Not sitting but PAV free	0.104 (0.0278)	0.073	0.135	Beta	Chambers et al. [36]
Sitting independently	0.115 (0.0227)	0.081	0.150	Beta	Chambers et al. [36]
Walking independently	0.252 (0.0332)	0.176	0.328	Beta	Chambers et al. [36]
PAV	0.104 (0.0278)	0.073	0.135	Beta	Chambers et al. [36]
**Transition probabilities**^c^					
Not sitting and PAV free to death in SOC arm	0.0532	0.0372	0.0692	Beta	ENDEAR control arm
Not sitting and PAV free to death in treatment arm	0.0184	0.0129	0.0239	Beta	ENDEAR treatment arm
Not sitting and PAV free to PAV in SOC arm	0.0625	0.0437	0.0812	Beta	ENDEAR treatment arm
Not sitting and PAV free to PAV in SOC arm	0.0355	0.0248	0.0461	Beta	ENDEAR treatment arm
PAV to Death	0.0146	0.0102	0.0190	Beta	Gregoretti et al. [54]
Sitting independently to Death	*λ* = 0.0006 *p* = 1.9613	0.0017 1.7226	0.0002 2.2331	Beta	Zerres et al. [14]
Walking independently to Death	Australian population mortality rate	–	–	Beta	Australian population mortality rate

*PBS* Pharmaceutical Benefits Scheme, *MBS* Medicare Benefits Schedule, *NHCDC* National Hospital Cost Data Collection, *PAV* permanent assisted ventilation, *VFS* ventilation-free survival, *OS* overall survival

^a^The values are presented as integral numbers in the table

^b^The values are presented with three decimals. The standard deviations (SD) reported in the paper by Chambers et al. [36] were greater than the mean values by patients, which were inconsistent of the SDs reported by caregivers in the paper. Thus, the SDs used in our model were divided by ten as shown in Table 2

^c^The parameters are presented with four decimals in the table

### Measurement of costs

The model considered the direct medical costs associated with drug acquisition, administration and monitoring, and healthcare resource use in each health state (Table 2). The treatment costs of nusinersen and AVXS-101 were from the Pharmaceutical Benefits Scheme [21] and the market price, respectively. The costs of administration and monitoring for nusinersen and AVXS-101 were from the Pharmaceutical Benefits Scheme [47], the Medicare Benefits Schedule [48], and the National Hospital Cost Data Collection [49] in Australia. The health state costs of “not sitting and PAV free”, “sitting independently” and “walking independently” were derived from a recently published study in Australia, which captured the direct costs of Type I, Type II, and Type III SMA, respectively [36]. The health state costs of “PAV” were the sum of the health state costs of “not sitting and PAV free” [36] and the specific costs of PAV derived from the report by the CER Institute [30]. All costs were converted to 2020 values in Australian dollars using the CCEMG-EPPI-Centre converter [50] and results were presented as the incremental cost-effectiveness ratio (ICER).

### Transition probabilities

The transition probabilities were estimated using parametric survival modelling [51]. The published Kaplan–Meier curves were digitised using computer digitisation programs (Digitizelt software; version 2.5.3). The individual patient data were reconstructed using an algorithm by Guyot et al. [52]. This algorithm was written as an R function in 2012, which was then implemented using the statistical package Stata by Wei and Royston [53]. Thus, we used the Stata package to run the algorithm (StataCorp. 2017; Release 15; College Station, TX: StataCorp LLC.software). The parametric models were then fitted to the reconstructed individual patient data in the statistical package Stata. The best-fit distributions were selected based on the Akaike information criterion, the Bayesian information criterion, and the Cox- Snell residuals (Table S2 and Figs. S1–S4 in the supplementary materials). The survival curves are presented in the supplementary materials (Fig. S5).

The survival function and transition to “PAV” for patients with SMA Type I were derived from the overall survival and event-free survival curves [41] in the ENDEAR clinical trial [37]. The transition probability to “PAV” was the difference between the overall survival and event-free survival at each timepoint. Survival of “PAV” was derived from the non-invasive respiratory muscle aid arm in the published study [54]. Survival of patients with SMA Type II was obtained from the study by Zerres et al. [14]. Survival of patients with SMA Type III was based on the general survival of Australian population [55].

Patients with SMA Type I in the SOC arm cannot achieve improvement of motor milestone, instead, either death or deteriorating to “PAV”. Patients with SMA Type I in either treatment arm can achieve motor improvement. However, there is no long-term clinical evidence of improvement or relapse of both treatments. Thus, these patients were assumed to remain in the health states until death after the treatment duration. The proportions of patients in the nusinersen arm who were “sitting independently” in each interval were obtained from ENDEAR clinical trial [41]. No patients achieved “walking independently” in the nusinersen arm, and no patients achieved sitting or walking independently in the SOC arm [41, 56]. In the clinical trial of AVXS-101, no patients ended up with death or PAV. Two patients achieved “walking independently” and another nine achieved “sitting independently” [42, 57].

### Sensitivity analysis and threshold analysis

One-way sensitivity analyses were performed to identify the key drivers impacting on the ICERs, in which the parameters were varied with ±30% of the mean values. Probabilistic sensitivity analysis (PSA) was also performed to jointly explore the variations of the model parameters by sampling these parameters simultaneously from appropriate distributions. The input parameters and distributions for sensitivity analysis are displayed in Table 2. A threshold analysis was conducted to estimate the price of AVXS-101 of being cost-effective with the change of WTP thresholds.

## Results

### Base-case analysis

The base-case results are presented in Table 3. For AVXS-101 compared to SOC, incremental costs were $4,111,471, and increment QALYs were 2.27, resulting in an ICER of $1,808,471 per QALY. For nusinersen compared to SOC, incremental costs were $1,669,191, and incremental QALYs were 0.30, leading to an ICER of $2,772,798. For AVXS-101 compared to nusinersen, incremental costs were $2,442,280, and incremental QALYs were1.97, resulting in an ICER of $1,238,288 per QALY.

**Table 3:** Incremental cost-effectiveness ratios (ICERs) for base-case analysis

	Cost	QALY	Incremental cost	Incremental QALY	ICER (cost per QALY)
**Nusinersen vs SOC**					
SOC	$923,335	0.301	$1,669,191	0.301	$2,772,798
Nusinersen	$2,592,526	0.602			
**AVXS-101 vs SOC**					
SOC	$923,335	0.301	$4,111,471	2.273	$1,808,471
AVXS-101	$5,034,806	2.574			
**AVXS-101 vs nusinersen**					
Nusinersen	$2,592,526	0.602	$2,442,280	1.972	$1,238,288
AVXS-101	$5,034,806	2.574			

The costs are presented as integral numbers. The QALYs are presented with three decimal places. The ICERs were calculated based on the original values rather than the rounded values and were rounded to integer numbers as shown in the table

*QALYs* quality-adjusted life years gained, *SOC* standard of care

### Sensitivity analysis

In the one-way sensitivity analysis, for the comparisons that AVXS-101 was involved, the cost of using AVXS-101 had the strongest impact on the ICERs (Figs. S7 and S8). Utility values in sitting and walking independently were also important drivers of the ICERs for these two comparisons. In both comparisons with nussinersen (Figs. S6 and S8), cost of using nusinersen had a strong impact on both ICERs. Utility value in sitting independently also had an important impact for the comparisons with nusinersen.

**Fig. 2:**
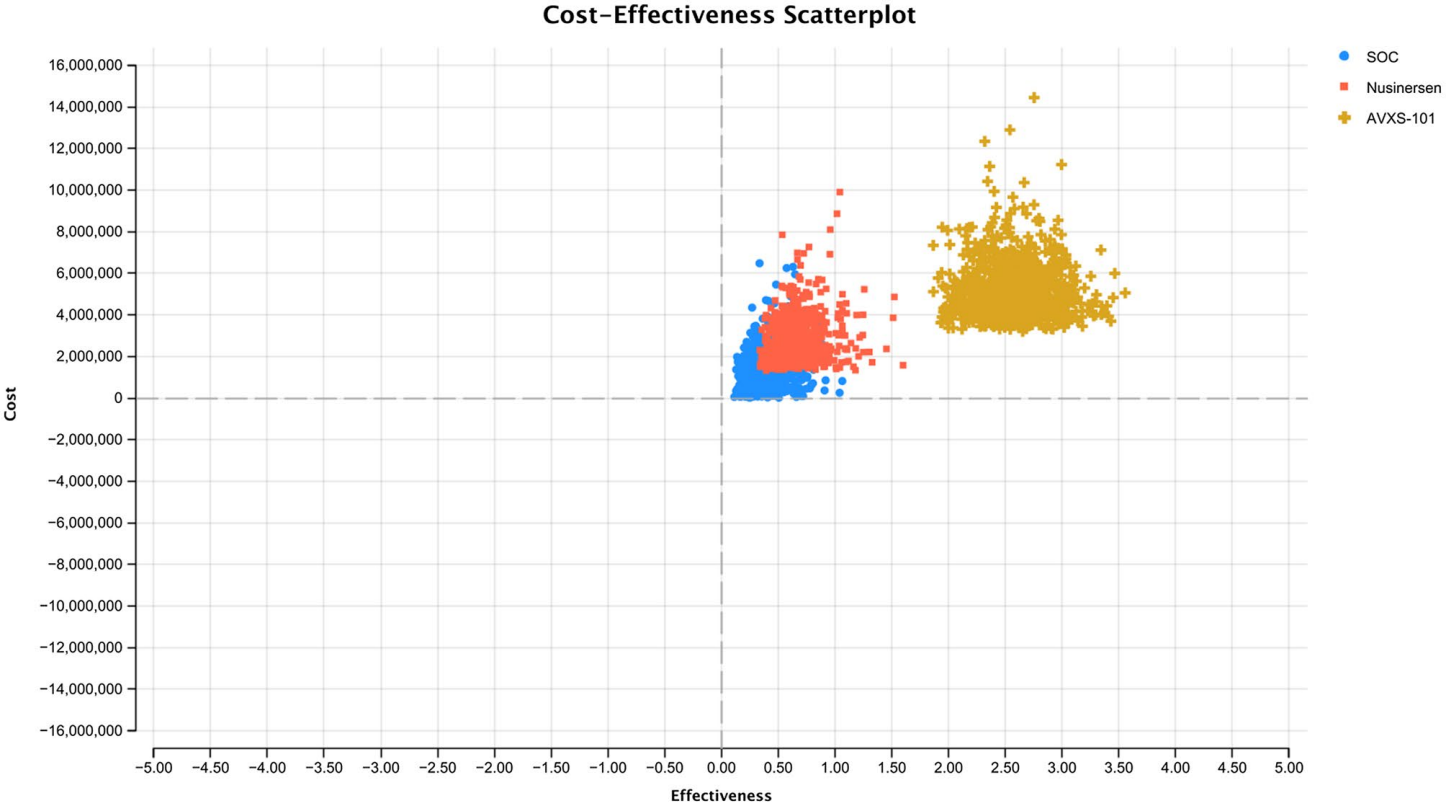
Cost-effectiveness scatterplot of AVXS-101, nusinersen and SOC

**Fig. 3:**
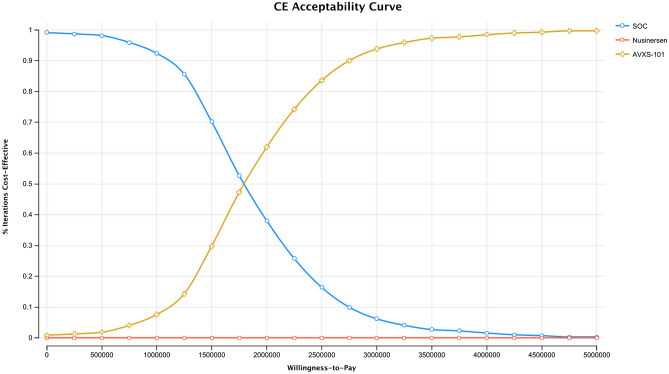
Cost-effectiveness acceptability curve of AVXS-101, nusinersen and SOC

The cost-effectiveness scatterplot of the three strategies (i.e., AVXS-101, nusinersen, and SOC) is presented in Fig. 2. The cost-effectiveness acceptability curve can be found in Fig. 3. Given a WTP threshold of $50,000 per QALY, the probability that AVXS-101 was cost-effective compared to SOC was 1.2%. Given a WTP threshold of $1,750,000 per QALY, the probability of cost-effectiveness for AVXS-101 compared to SOC was 52.8%. Given a WTP threshold of $6,800,000 per QALY, the probability that AVXS-101 was cost-effective compared to SOC reached 100%.

### Scenario analysis

With WTP thresholds of less than $500,000 per QALY, AVXS-101 would not be cost-effective compared to SOC. With a WTP threshold of $500,000 per QALY, AVXS-101 would be cost-effective with a price of $79,599. With a WTP threshold of $1,000,000 per QALY, AVXS-101 would be cost-effective with a price of greater than one million dollars (Fig. S9 in the supplementary materials).

## Discussion

Based on this cost-effectiveness analysis for the treatments of patients with SMA Type I, both AVXS-101 and nusinersen were not cost-effective using a threshold of $50,000 per QALY. Although the cost-effective evidence for reimbursement policies in this study is provided at a country-level basis, the analysis of the treatments on the ICERs will be comparable in other countries.

In the economic evaluations identified by the systematic review [28], only one compared AVXS-101 against nusinersen, which showed that AVXS-101 was cost-saving at a price of about $3.5 million [16]. The results by the CER Institute reported that the ICERs of nusinersen and AVXS-101 compared to SOC, respectively were about $1.6 million per QALY and $350,000 per QALY [30]. One paper funded by the manufacturer of nusinersen reported an ICER of approximately $782,000 per QALY [10] and the ICER of the manufacturer’s submission to the Canadian authority was about $956,000 per QALY [29]. The Canadian authority considered that the model and assumptions were highly optimistic, and a re-analysed result was around $13 million per QALY [29].

The different ICERs of all these studies can be attributed to the quality of clinical evidence. SMA is a rare disease, thus, the sample sizes used in clinical trials were small and likely to continue to be small, such as the total 15 patients in the clinical trial of AVXS-101 [42]. Although ENDEAR included large patients (i.e., 121) given the rarity of the condition, the uncertainty is that these treatments may offer long-term benefits which however could not be captured in the trials. The lack of long-term clinical data makes it difficult to provide strong and accurate cost-effectiveness evidence. Patients treated with nusinersen may achieve walking in the follow-up, which no peer-reviewed publication reported. Additionally, SMA is a life-threatening condition. Patients with SMA Type I in SOC arm will be rapidly progressed, thus long-term randomised control trials may be unethical to be conducted.

The different ICERs of all these economic evaluations can also be attributed to the measurement of utility values which varied considerably among the existing literature (Table 1) [16, 44, 46]. One of the most important reasons for the large inconsistencies in measuring utility value of children/infants is that the commonly used approach is to have parents or guardians (i.e., proxy) respond to questionnaires about their child. This approach is pragmatic, but proxy versions of instruments and responses might not accurately reflect the utility values of infants. In addition, the utility values for patients with SMA Type III used in the report by the CER institute [30] were derived from the utility values of the general population in the US. However, patients with this type still have symptoms and need disease management for SMA. Thus, using the utility values of the general population for patients with SMA Type III would be highly optimistic for “walking independently” health state, leading to more cost-effective conclusions. Given the considerable variations in utility values, more conservative values were used in our study. Additionally, both nusinersen and AVXS-101 are still new interventions for treating SMA patients, thus long-term clinical evidence is unclear and using conservative estimates is reasonable.

The utility values reported for Australian population in the literature [36] were not increased as motor milestones improved; this resulted in the utility value of SMA Type II being lower than that of Type I. This could be explained that the recruited patients might not be representative for the patients with SMA Type II. For example, as reported in the study [36], nine of the recruited patients with SMA Type II (37.5%) were not able to sit independently, which however, typical patients with SMA Type II could achieve sitting independently in general [2]. Therefore, in our study, we used the average utility value of patients with SMA from Type I to Type III for patients who achieved “sitting independently” in the model (Table 2), which value was greater than that of Type I but less than that of Type III.

Compared to SOC, using nusinersen generated a higher ICER than using AVXS-101 in our model. It should be noted that the price of nusinersen used in this study was the listed price in the Pharmaceutical Benefits Scheme. The negotiated price of nusinersen is confidential. AVXS-101 has been approved by Therapeutic Goods Association but deferred by Pharmaceutical Benefits Advisory Committee. The negotiated price of AVXS-101 is uncertain in Australia. Thus, it remains unknown whether nusinersen is dominated by AVXS-101 with the negotiated price of nusinersen and the future decisions of listing AVXS-101.

Using the current price, AVXS-101 was not cost-effective under the commonly used threshold of $50,000 per QALY. Other commonly used thresholds include a range of £20,000 to £30,000 in the UK and the thresholds of one to three times the gross domestic product (GDP) per capita in the low- and middle-income countries [58]. However, for rare diseases such as SMA, decision-making on reimbursement is challenging because many rare diseases are extremely costly to manage [59] and treatments for rare diseases are less cost-effective than treatments for more common diseases. For example, the range of ICERs for treating Fabry, Gaucher, and Pompe diseases were reported from hundreds/thousands of dollars to several millions of dollars per QALY [60]. Therefore, decision-makers face the dilemma of whether to fund drugs that would not normally be considered cost-effective. Some viewed that the cost-effectiveness of drugs for rare diseases should be treated the same way as for other diseases and adhere to the standard thresholds [61, 62]. However, others argued that when making coverage decisions, we should consider multiple factors such as clinical, economic, and ethical issues [63]. When dealing with rare diseases, policy makers face other issues such as compassion and beneficence in their decision-making which in turn leads to difficulties in applying cost-effectiveness analysis and thresholds [63].

Although economic evaluations use thresholds to provide economic evidence on reimbursement, it is not a one-size-fits-all approach. In Australia, there is no fixed threshold. To access the value-based preference of publicly funding pharmaceuticals by Pharmaceutical Benefits Advisory Committee, one study analysed the funding decisions from 1994 to 2009 in Australia [64]. The results showed that the drugs which had ICERs greater than $100,000 per QALY rarely received positive decisions for funding by the Pharmaceutical Benefits Advisory Committee. If a condition was both life-threatening and there was no effective treatment, the new drug was more likely to receive a positive decision, which was equivalent to a reduction of $46,000 in cost per QALY [64]. However, for SMA, this reduction could not offset the gap between $100,000 per QALY and the ICERs of AVXS-101 or nusinersen in our model. In 2021, AVXS-101 was approved by the Therapeutic Goods Association but deferred by the Pharmaceutical Benefits Advisory Committee [25, 26]. On the other hand, nusinersen is currently recommended for reimbursement by the Pharmaceutical Benefits Advisory Committee, even if the ICER of nusinersen compared to SOC was more than $2 million per QALY in our model. Given the complexity of rare diseases, it is valuable to explore the threshold for such diseases.

## Conclusion

With a threshold of $50,000 per QALY, neither AVXS-101 nor nusinersen were cost-effective compared to SOC. With a WTP threshold of $500,000 per QALY, AVXS-101 would be cost-effective with a price of $79,599. The key drivers of influencing ICERs were the costs of using these treatments, and utility values of sitting and walking independently. Either decreasing the costs or increasing the utility values could substantially make both interventions more cost-effective compared to SOC. The limited evidence from clinical trials is one of the most important concerns for developing economic models. Exploring appropriate WTP thresholds for rare diseases is also critical for making reimbursement decisions in a publicly funded healthcare system.

## Supplementary Information

Below is the link to the electronic supplementary material.

Supplementary file1 (DOCX 23 KB)

Supplementary file2 (DOCX 1407 KB)
