# Supplementary material for: Cost-effectiveness analysis of gene-based therapies for patients with spinal muscular atrophy type I in Australia
Source: J Neurol. 2022 Aug 18;269(12):6544–54. doi: 10.1007/s00415-022-11319-0 (PMC9618547; doi:10.1007/s00415-022-11319-0)
Supplement: Supplementary file 2 — Supplementary file2 (DOCX 1407 KB) [file 415_2022_11319_MOESM2_ESM.docx]

**TITLE**

Cost-Effectiveness Analysis of Gene-based Therapies for Patients with Spinal Muscular Atrophy Type I in Australia

**JOURNAL NAME**

Journal of Neurology

**FULL NAMES AND AFFILIATION**

**Tianjiao Wang^1,2^, Paul Scuffham^1,2^, Joshua Byrnes^1,2^, Martin Downes^1,2^**

**^1^** Centre for Applied Health Economics, School of Medicine and Dentistry, Griffith University, Brisbane, Queensland, Australia

**^2^** Menzies Health Institute Queensland, Griffith University, Gold Coast, Queensland, Australia

**CORRESPONDING AUTHOR**

**Tianjiao Wang**

tianjiao.wang@griffithuni.edu.au

Phone: +61 7 3735 3251

**Supplementary Materials**

[**Table S1 Key assumptions used in the model** 3](#_Toc104905025)

[**Table S2 Fitting distributions using Akaike information criterion and Bayesian information criterion** 5](#_Toc104905026)

[**Figure S1 Fitting distributions using Cox-Snell residuals for SMA Type I overall survival** 6](#_Toc104905027)

[**Figure S2 Fitting distributions using Cox-Snell residuals for SMA Type I ventilation-free survival** 7](#_Toc104905028)

[**Figure S3 Fitting distributions using Cox-Snell residuals for permanent assisted ventilation overall survival** 8](#_Toc104905029)

[**Figure S4 Fitting distributions using Cox-Snell residuals for SMA Type II overall survival** 9](#_Toc104905030)

[**Figure S5 Survival curves used in the analysis** 11](#_Toc104905031)

[**Figure S6 Tornado diagram for one-way sensitivity analysis of nusinersen versus SOC** 12](#_Toc104905032)

[**Figure S7 Tornado diagram for one-way sensitivity analysis of AVXS-101 versus SOC** 13](#_Toc104905033)

[**Figure S8 Tornado diagram for one-way sensitivity analysis of AVXS-101 versus nusinersen** 14](#_Toc104905034)

[**Figure S9 Costs of AVXS-101 with the variations of WTP thresholds** 15](#_Toc104905035)

# **Table S1 Key assumptions used in the model**

| **Assumption** | **Rationale** |
| --- | --- |
| Once patients transition to another health states, they could not transition back to their previous health states in the next cycle. And only patients in “not sitting but PAV free” can transition to “PAV”. | Based on the published literature, patients who achieved motor milestones were hardly to be transitioned to a worse health state of “PAV”. This assumption is consistent with the published literature. |
| In the treatment arms, patients will remain in the health states that they reached at the end of the trials. | Treated patients could be expected to achieve higher motor milestone after the trials. However, no long-term clinical evidence of both treatments is known. Thus, patients are assumed to remain in the same health states until death. |
| When patients achieved improved motor milestones of sitting, they will have the same survival as patients with SMA Type II. | Patients with similar symptoms and disease burden will likely have similar survival. This assumption is consistent will the published studies. |
| When patients achieved improved motor milestones of walking, they will have the same survival as general population. | Published studies have reported that patients with Type III can have life expectancy close to the general population. |
| The average utility value of all types (I, II, and III) reported in the study by Chambers et al., 2020 in the Australian context was used for the patients with SMA Type II (i.e., “sitting independently” in the model). | The utility values used in the model were derived from the published information in Australia. The reported utility value of SMA Type II was 0.067 which was less than SMA Type I. This is not consistent with the previously published studies. More discussion can be found in the discussion part of the manuscript. |
| Nusinersen will be used continuously for patients until 2 years old. | The life expectancy of patients with spinal muscular atrophy (SMA) type I is generally considered to be less than 2 years. |

PAV: permanent assisted ventilation; SMA: spinal muscular atrophy

# **Table S2 Fitting distributions using Akaike information criterion and Bayesian information criterion**

|  |  | **Exponential** | **Weibull** | **Gompertz** | **Lognormal** | **Loglogistic** | **General Gamma** |
| --- | --- | --- | --- | --- | --- | --- | --- |
| **SMA Type I OS** | **AIC** | 101.388* | 102.6617 | 99.93045 | 99.7513* | 101.2753 | 98.40351 |
|  | **BIC** | 103.1016* | 106.0888 | 103.3576 | 103.1785* | 104.7024 | 98.40351 |
| **SMA Type I VFS** | **AIC** | 118.0515* | 119.9947 | 118.7273 | 114.7305 | 115.6458* | 114.9512 |
|  | **BIC** | 119.7651* | 123.4218 | 122.1544 | 118.1576 | 119.073* | 120.0919 |
| **PAV OS** | **AIC** | 94.84174* | 96.41549 | 96.6023 | 97.19599* | 96.87442 | 98.41499 |
|  | **BIC** | 96.27573* | 99.28346 | 99.47027 | 100.064* | 99.74239 | 102.7169 |
| **SMA Type II OS** | **AIC** | 566.5536* | 487.6869* | 494.0632* | 502.0288* | 498.32* | 488.9528* |
|  | **BIC** | 570.0175* | 494.6146* | 500.9908* | 508.9564* | 505.2476* | 499.3443* |

OS: overall survival; VFS: ventilation-free survival; PAV: permanent assisted ventilation; AIC: Akaike information criterion; BIC: Bayesian information criterion

* The parameters of the distributions have a p value < 0.05. Only the distributions with significant parameters were analysed using Cox-Snell residuals.

# **Figure S1 Fitting distributions using Cox-Snell residuals for SMA Type I overall survival**

| **Exponential** | **Lognormal** |
| --- | --- |
| **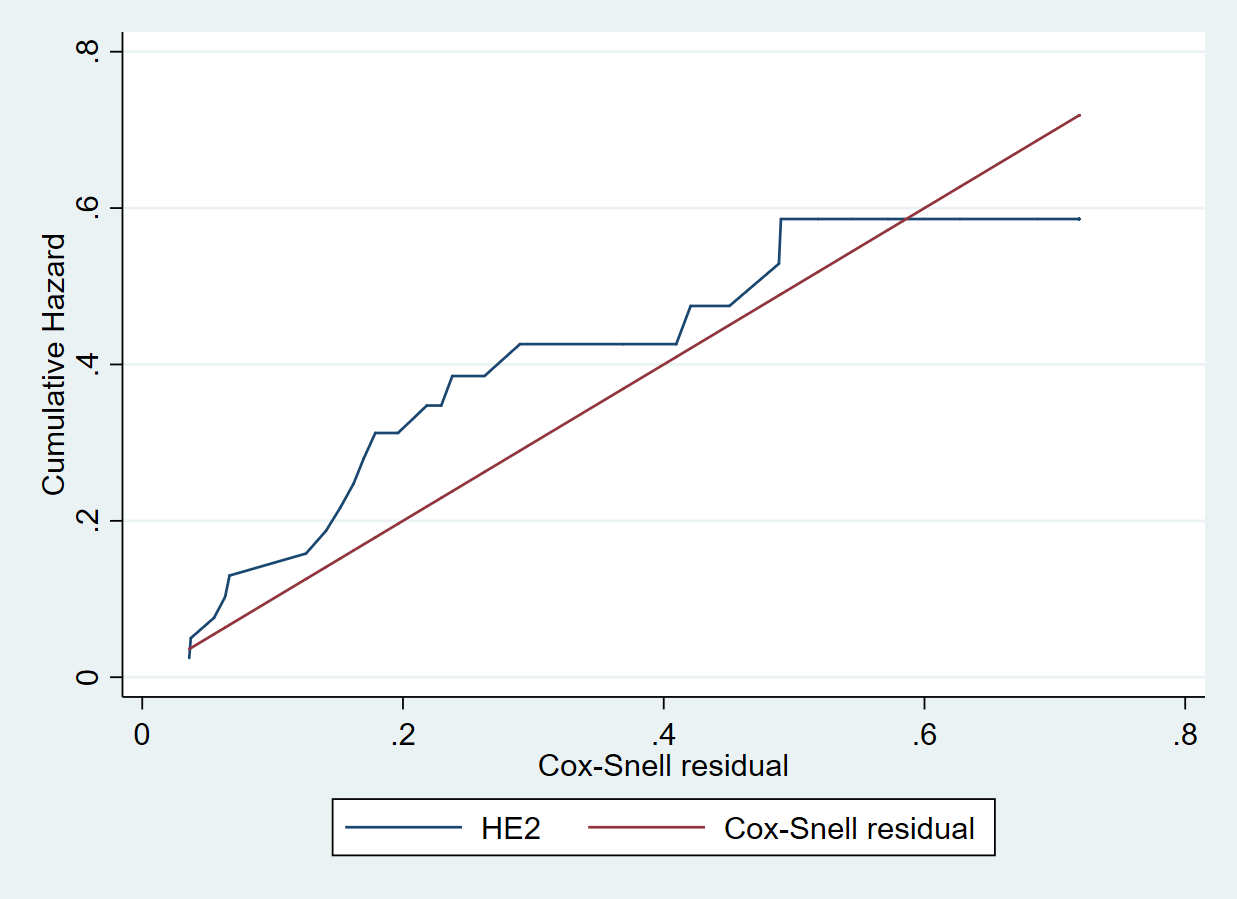** | **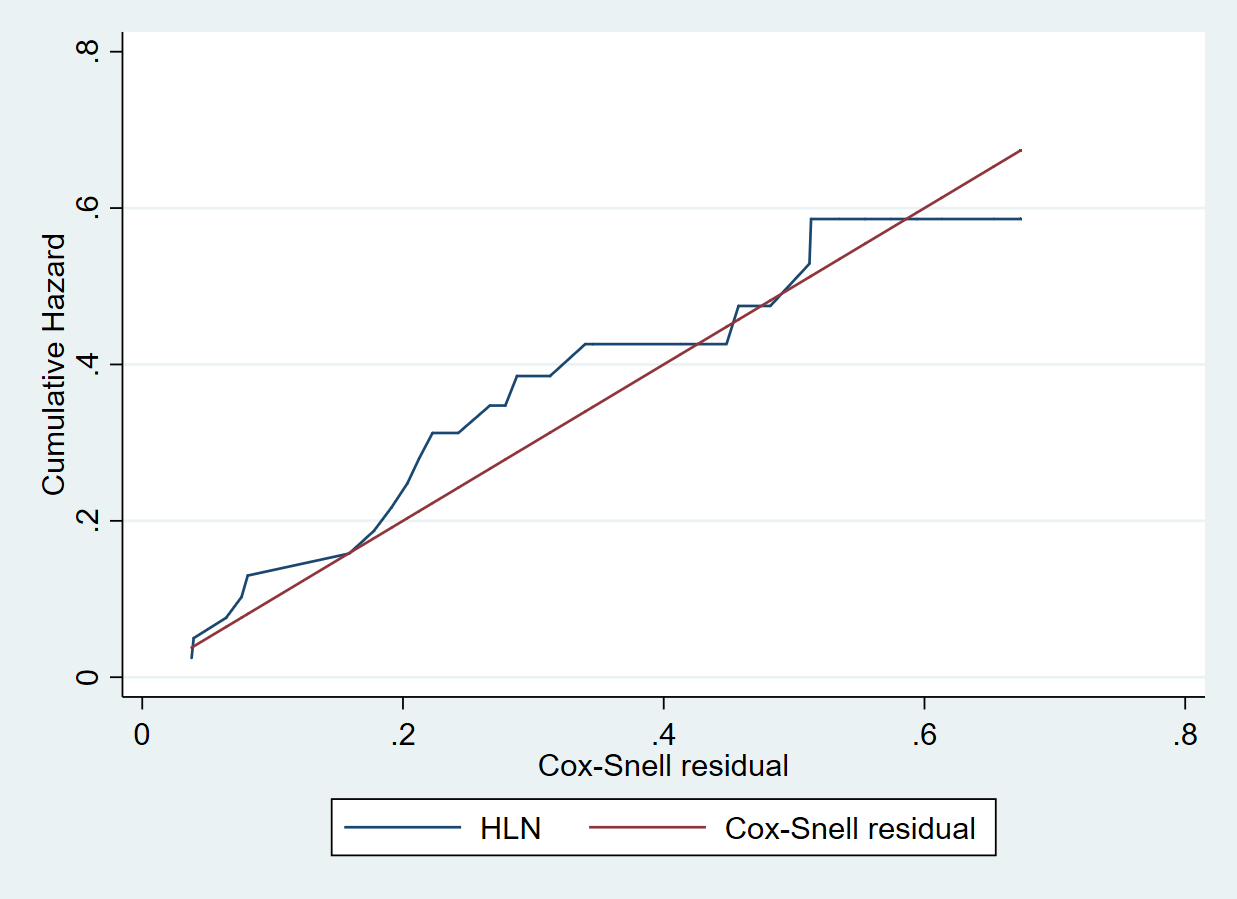** |

# **Figure S2 Fitting distributions using Cox-Snell residuals for SMA Type I ventilation-free survival**

| **Exponential** | **Log-logistic** |
| --- | --- |
| **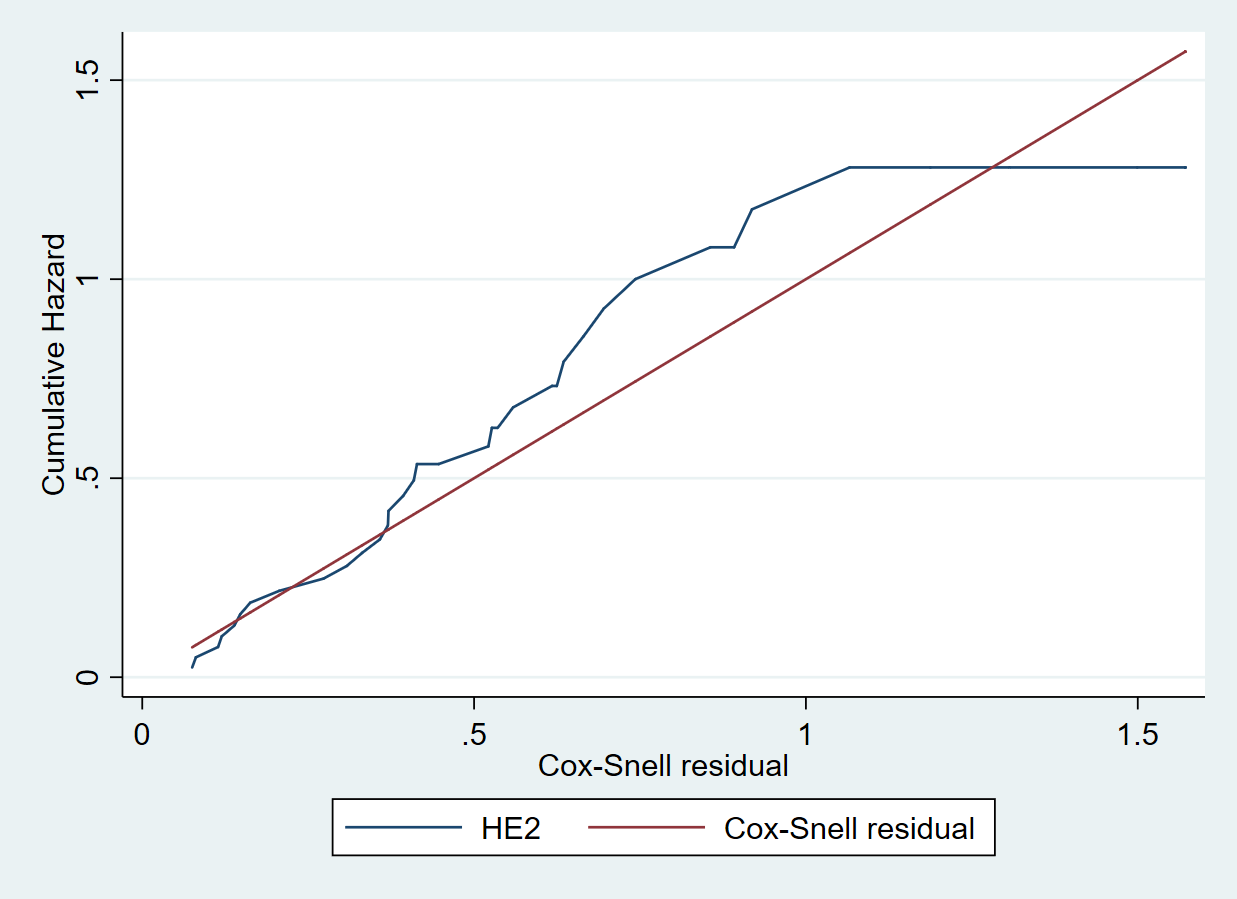** | **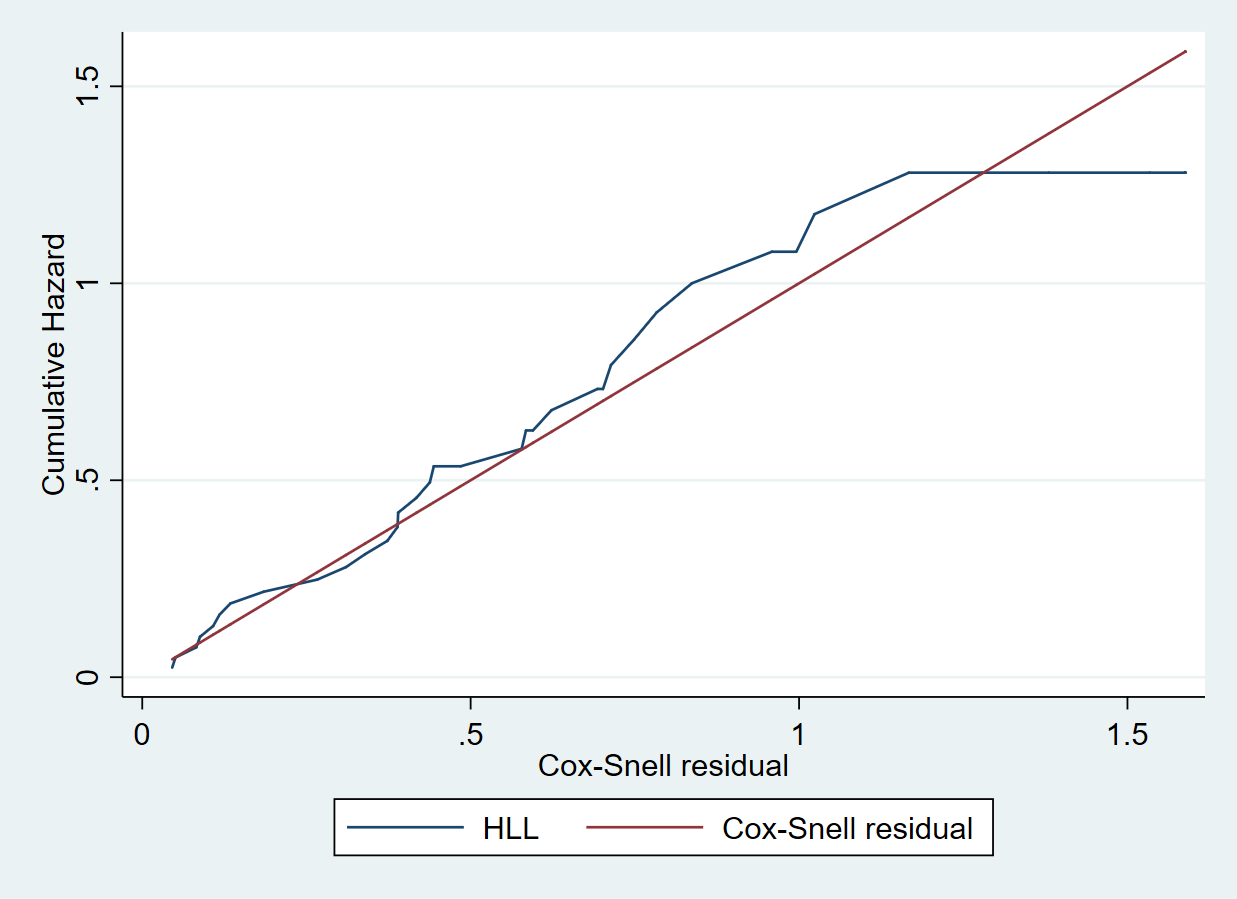** |

# **Figure S3 Fitting distributions using Cox-Snell residuals for permanent assisted ventilation overall survival**

| **Exponential** | **Lognormal** |
| --- | --- |
| **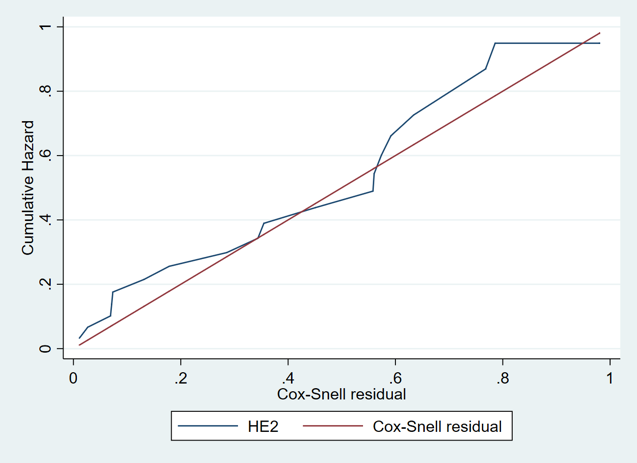** | 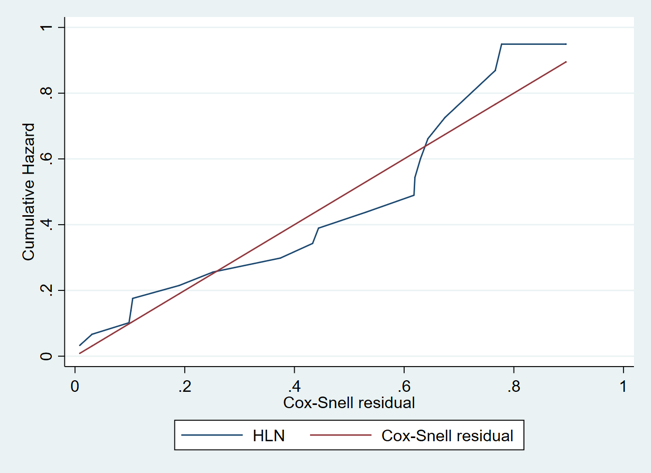 |

# **Figure S4 Fitting distributions using Cox-Snell residuals for SMA Type II overall survival**

| **Exponential** | **Weibull** |
| --- | --- |
| **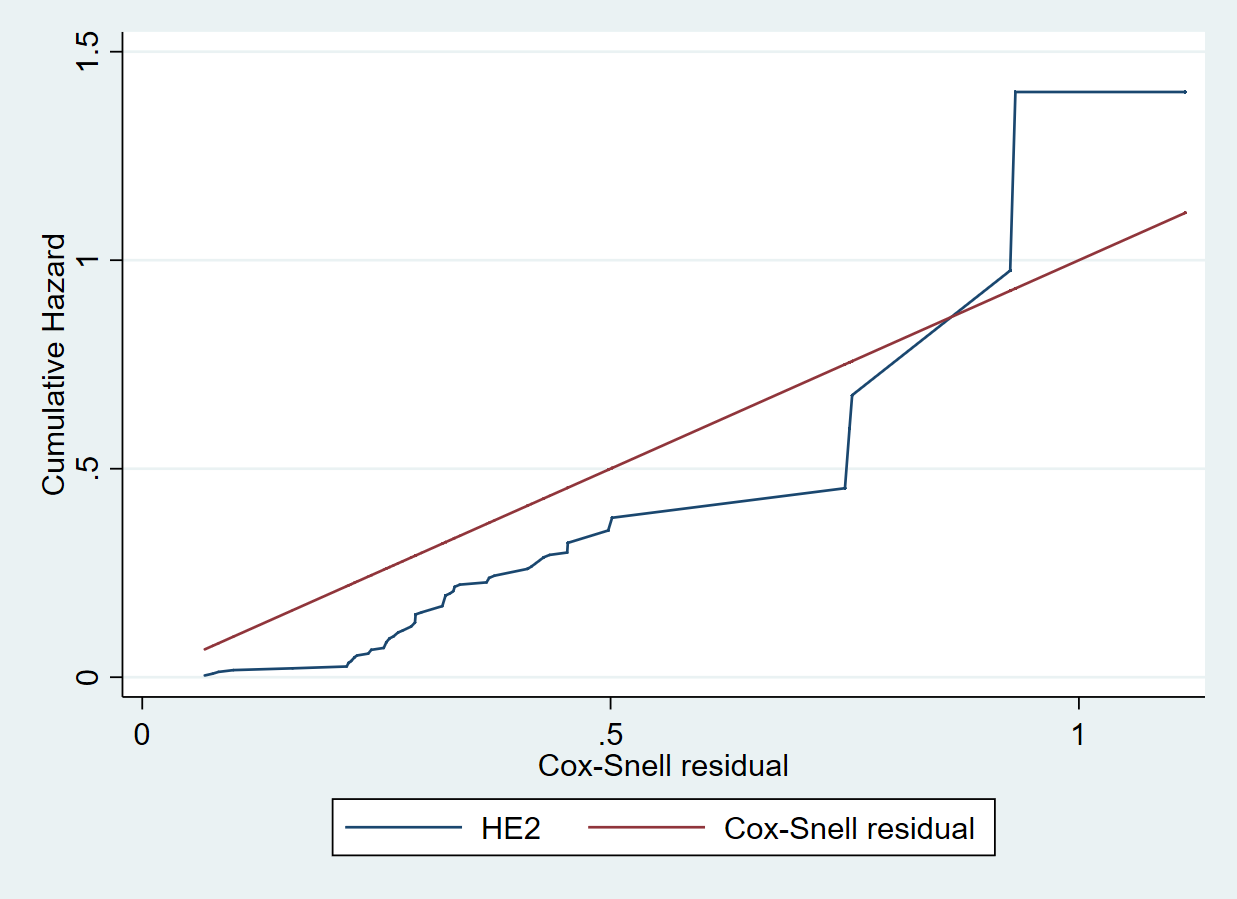** | 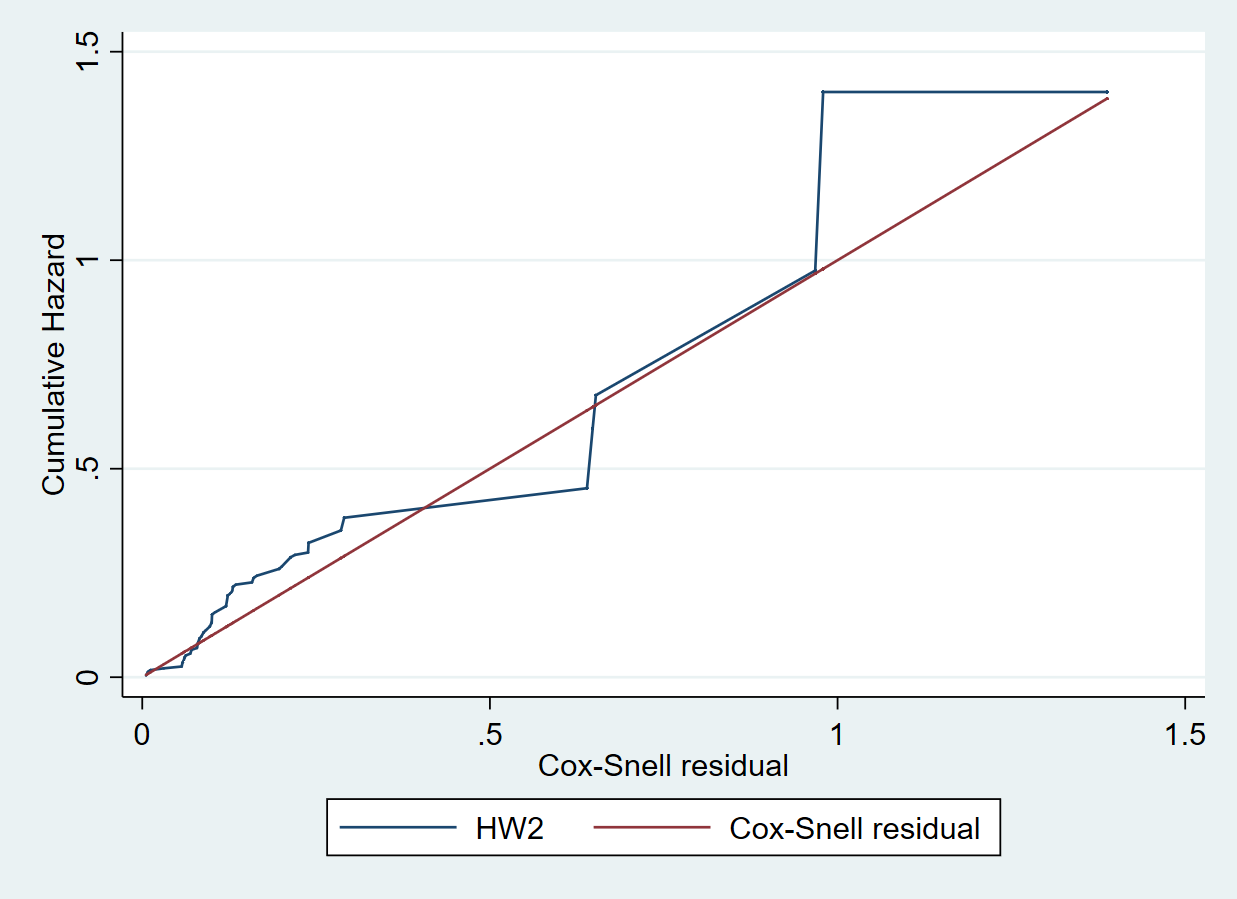 |
| **Gompertz** | **Lognormal** |
| 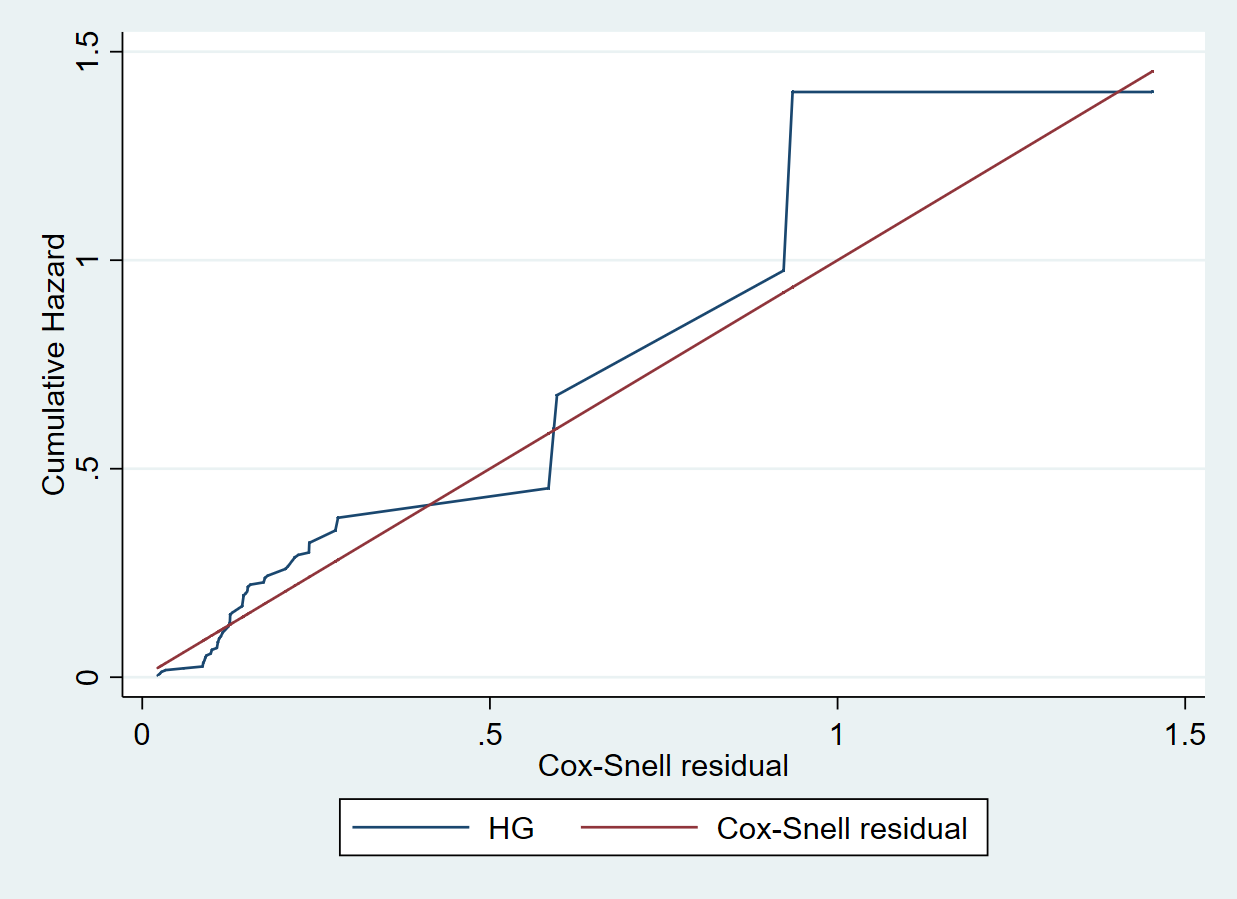 | 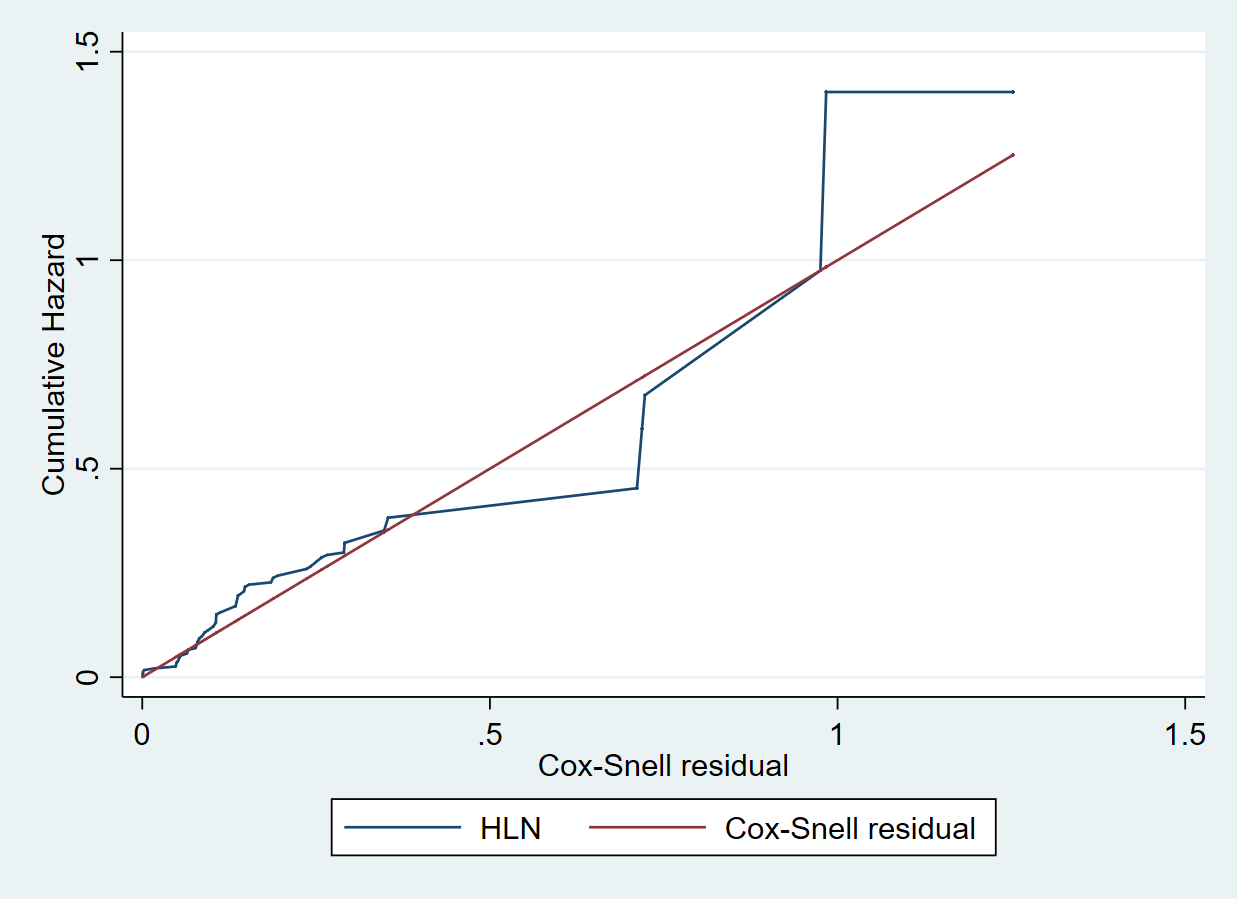 |
| **Log-logistic** | **General Gamma** |
| 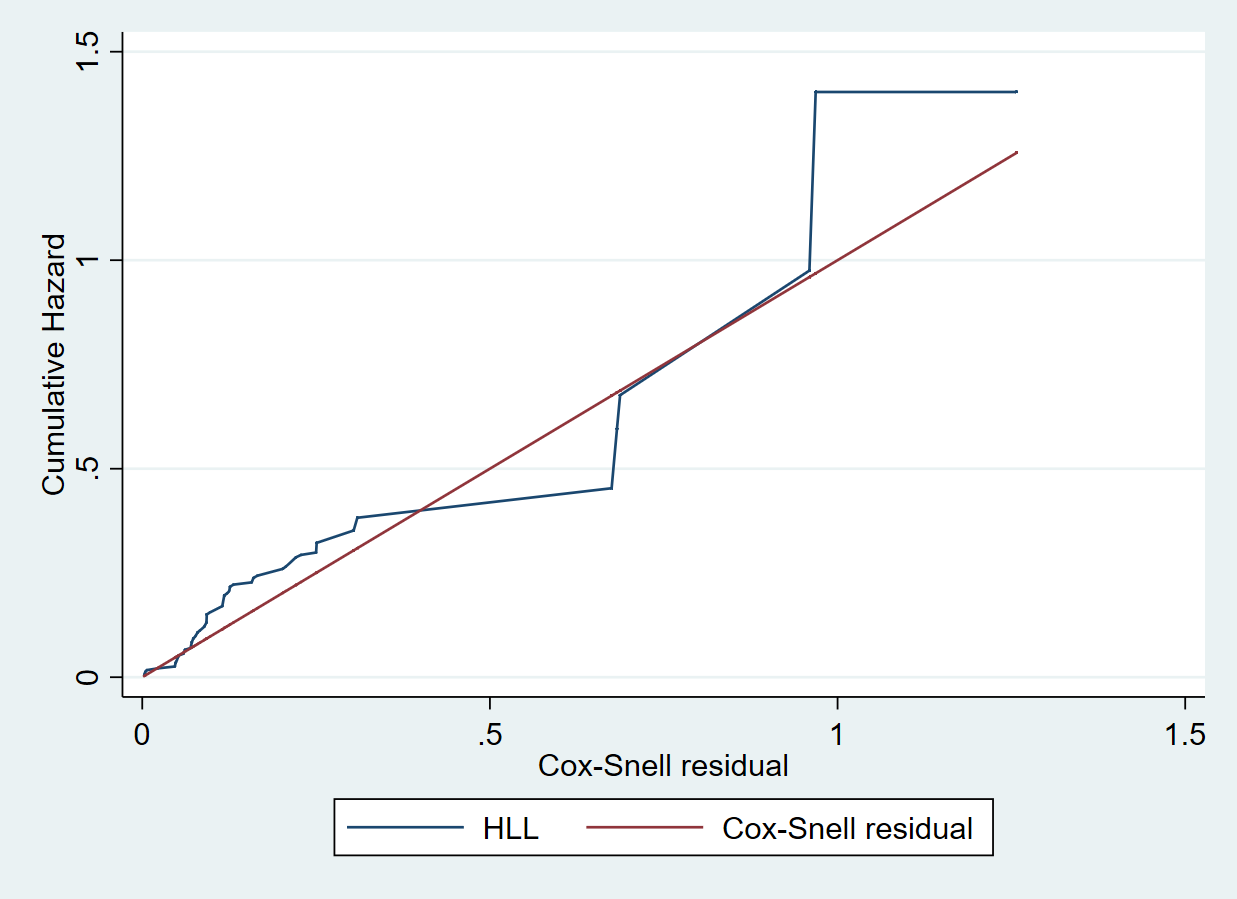 | 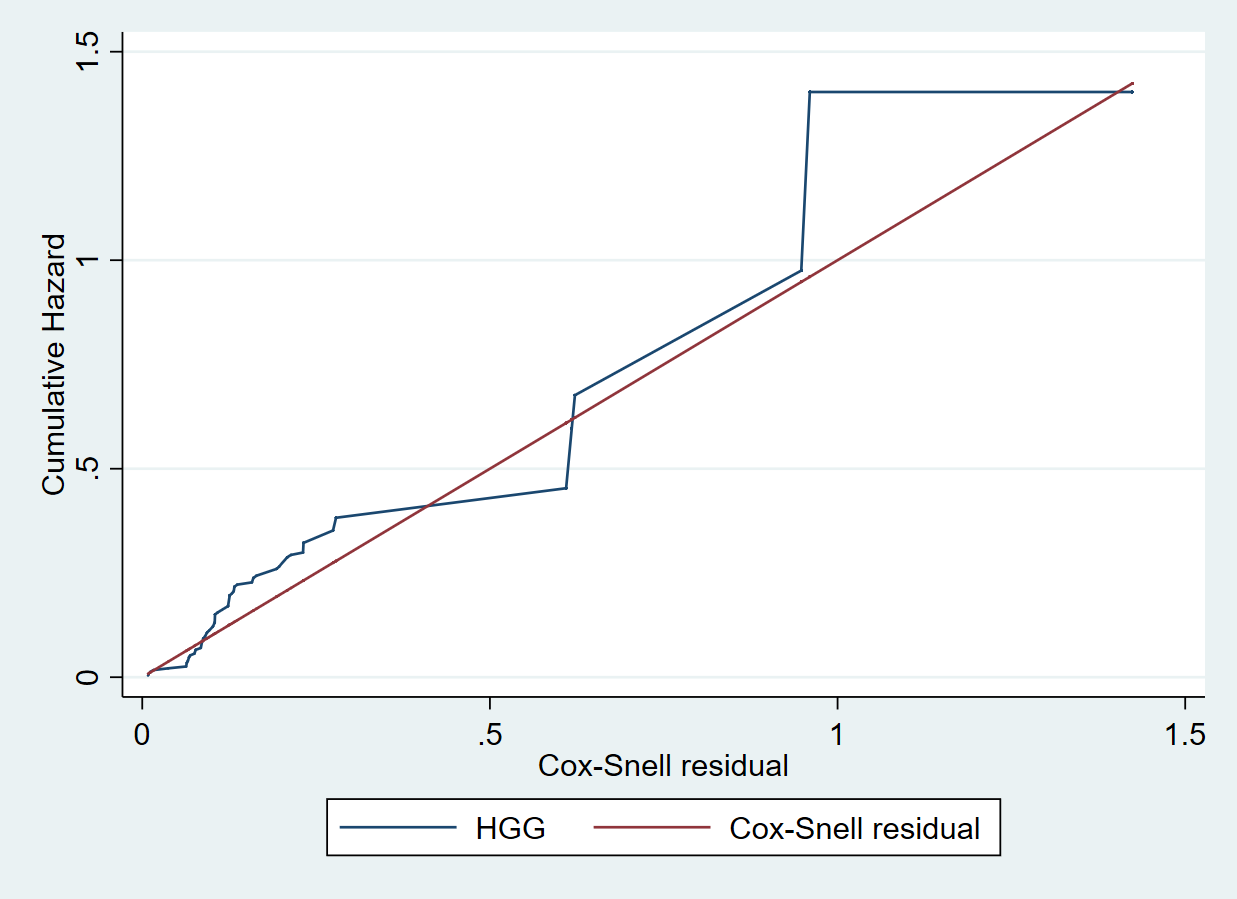 |


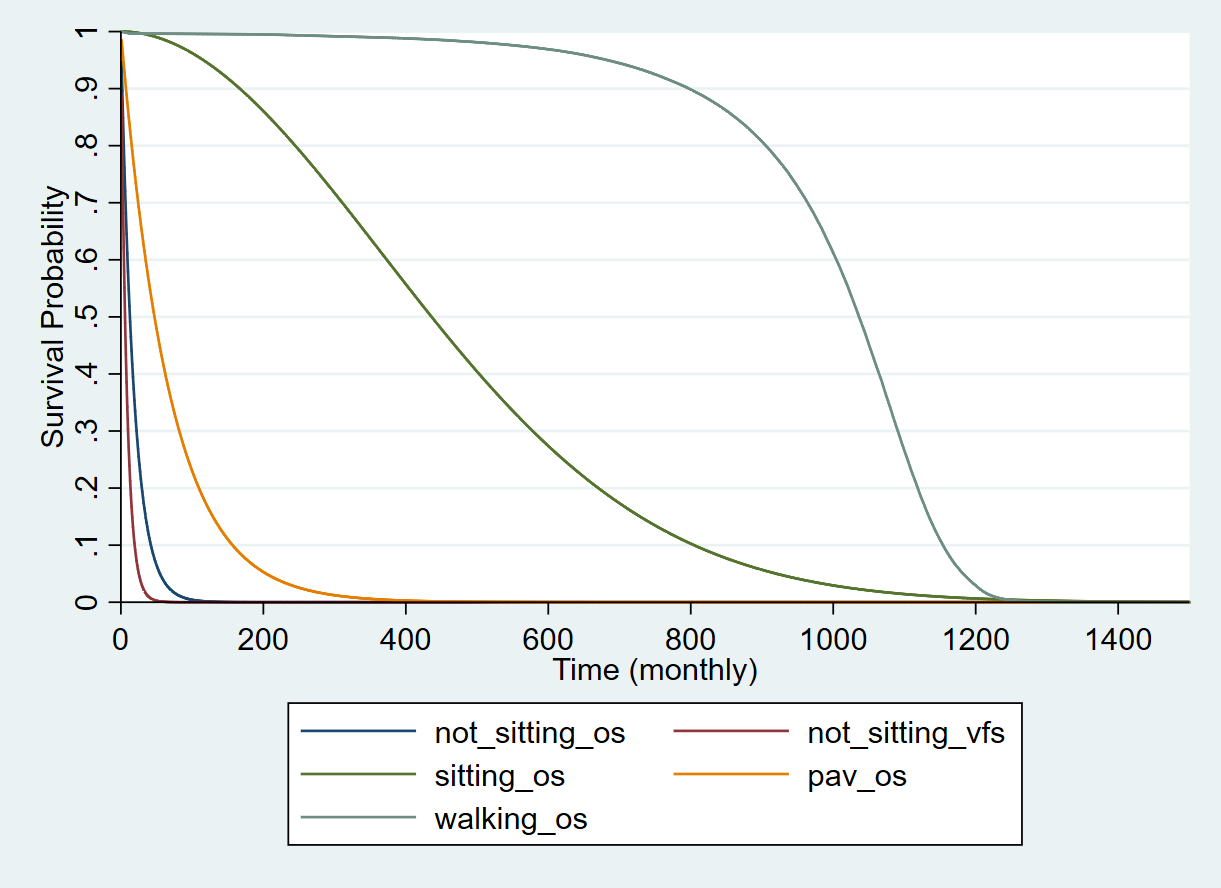


# **Figure S5 Survival curves used in the analysis**

OS: overall survival; PAV: permanent assisted ventilation; VFS: ventilation-free survival


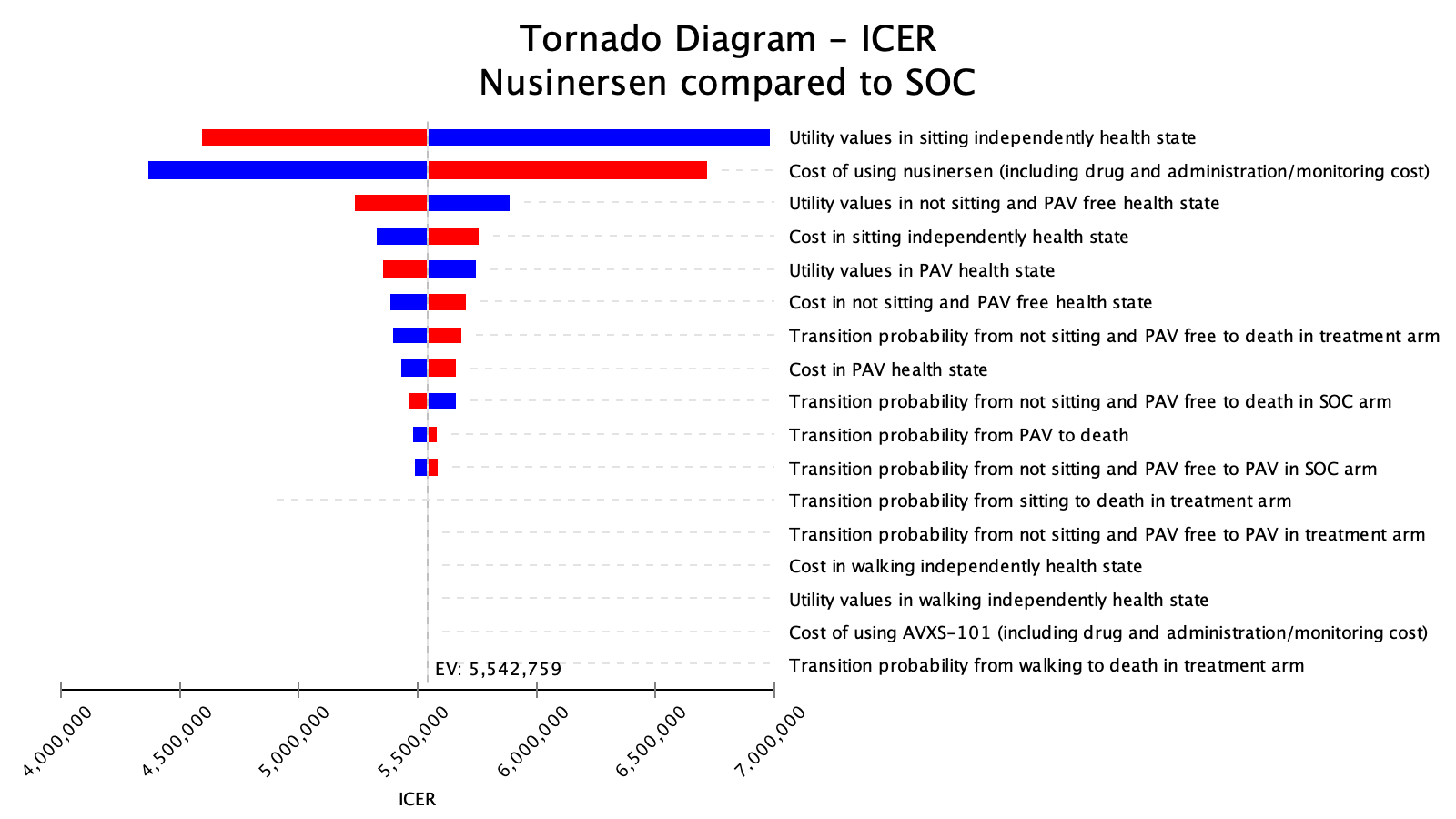


# **Figure S6 Tornado diagram for one-way sensitivity analysis of nusinersen versus SOC**

ICER: Incremental cost-effectiveness ratio; PAV: permanent assisted ventilation; SOC: standard of care

**
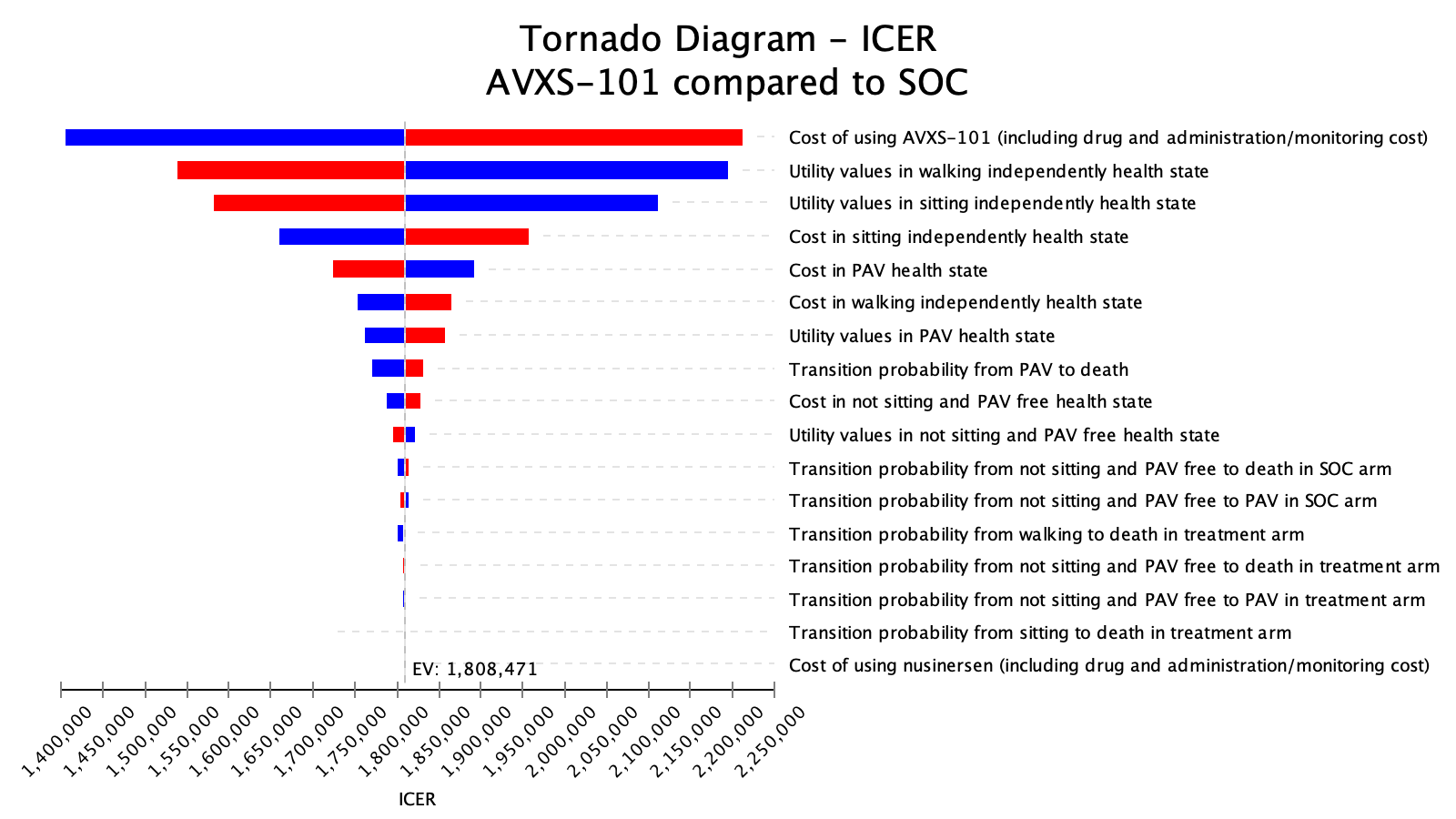
**

# **Figure S7 Tornado diagram for one-way sensitivity analysis of AVXS-101 versus SOC**

ICER: Incremental cost-effectiveness ratio; PAV: permanent assisted ventilation; SOC: standard of care


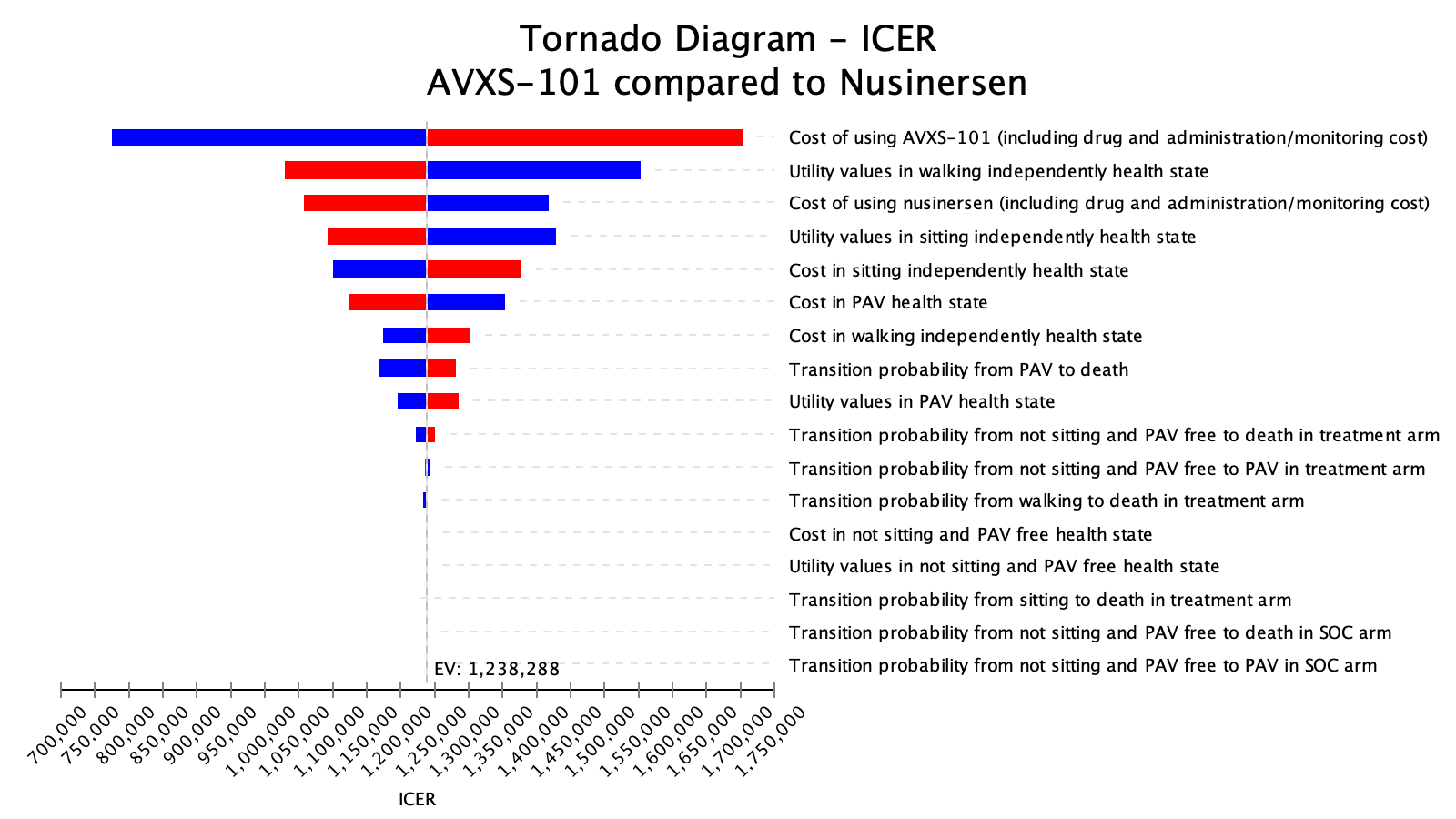


# **Figure S8 Tornado diagram for one-way sensitivity analysis of AVXS-101 versus nusinersen**

ICER: Incremental cost-effectiveness ratio; PAV: permanent assisted ventilation; SOC: standard of care


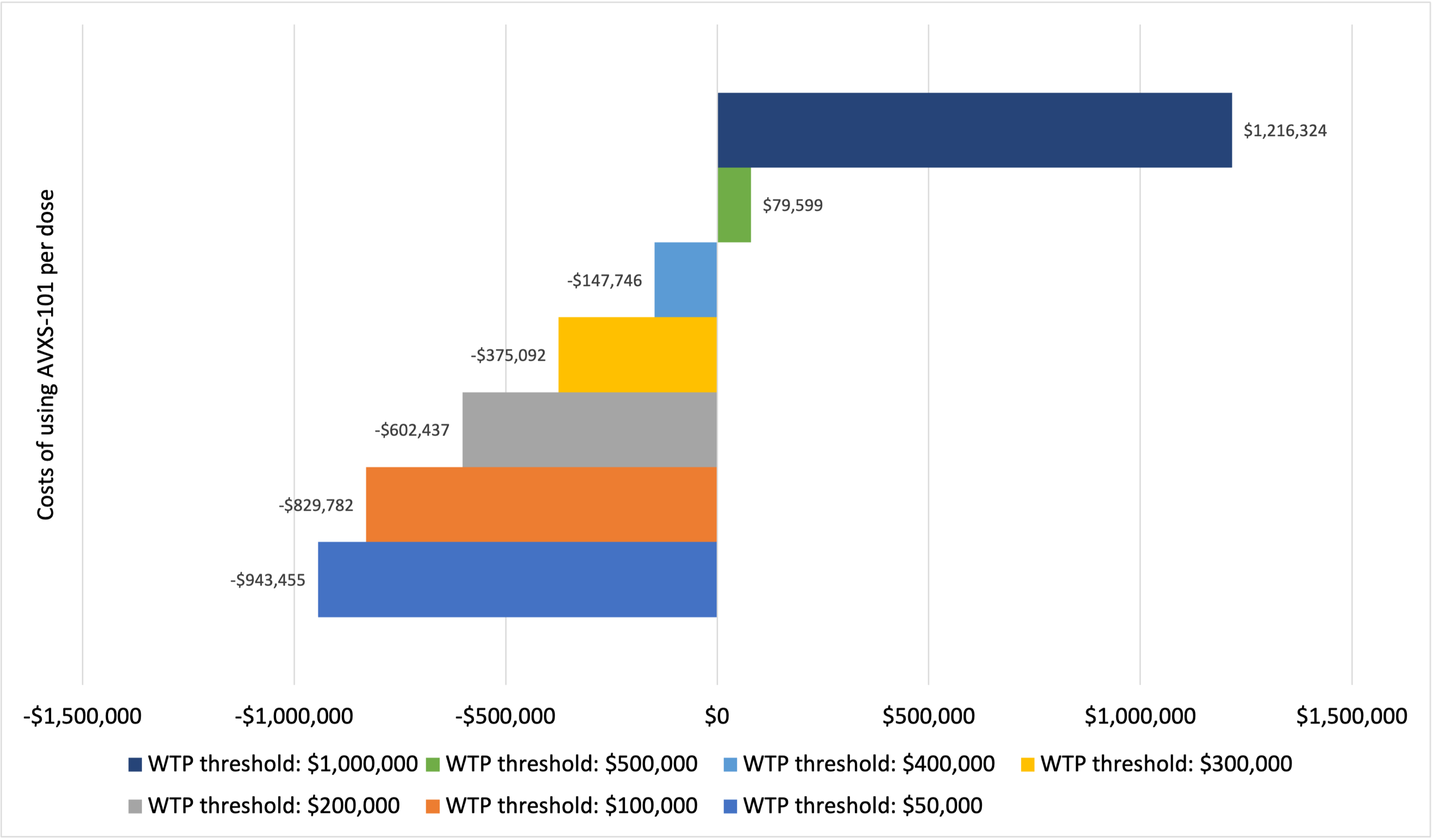


# **Figure S9 Costs of AVXS-101 with the variations of WTP thresholds**

WTP: willingness-to-pay
